# Tracking Recovery After TKA with Instrumented Timed Up and Go: Spatiotemporal, Rotational, and Strength Adaptations over Six Months

**DOI:** 10.3390/s26206585

**Published:** 2026-10-17

**Authors:** Elina Gianzina, Christos K. Yiannakopoulos, Andreas Bourantanis, Elias Armenis, Georgios Kalinterakis, Efstathios Chronopoulos, Elissavet Rousanoglou

**Affiliations:** 1 School of Physical Education and Sport Science, National and Kapodistrian University of Athens, 17232 Athens, Greece; 2 Laboratory for Research of the Musculoskeletal System “Th. Garofalidis”, Medical School, National and Kapodistrian University of Athens, KAT General Hospital, Kifissia, 14561 Athens, Greece

**Keywords:** knee osteoarthritis, total knee arthroplasty, timed up and go, gait analysis, IMU sensors, muscle strength, KOOS

## Abstract

**Highlights:**

**What are the main findings?**

- Significant overall changes in gait performance, muscle strength, and composite Knee Injury and Osteoarthritis Outcome Score were observed within 6 months after total knee arthroplasty.
- Phase-specific instrumented Timed Up and Go analysis identified different longitudinal patterns across functional mobility parameters, with significant changes in overall and walking-phase performance, while several turning-related measures showed no significant change over time.

**What are the implications of the main findings?**

- Instrumented Timed Up and Go enables sensitive detection of functional recovery patterns.
- Rehabilitation may benefit from targeting rotational stability and proprioceptive control.

**Abstract:**

Knee osteoarthritis (KOA) is a leading cause of disability, and total knee arthroplasty (TKA) improves pain and functional mobility, although gait impairments may persist postoperatively. This study investigated longitudinal changes in patient-reported outcomes, muscle strength, and gait performance using an instrumented Timed Up and Go (iTUG) test with wearable inertial measurement units (IMUs). Twenty-two patients with end-stage KOA were assessed preoperatively and at 3, 12, and 24 weeks postoperatively. The Knee Injury and Osteoarthritis Outcome Score five-subscale composite (KOOS5) showed a significant overall change over time (*p* < 0.001), while isometric muscle strength showed significant overall changes across all tested muscle groups (*p* ≤ 0.008), with mean values demonstrating an initial postoperative decline followed by subsequent increases. Total TUG duration showed a significant overall time effect (*p* = 0.023), with significant reductions observed in the forward and return gait phases (*p* = 0.012 and *p* = 0.001, respectively). End-turning maximum rotational speed and sit-to-stand vertical acceleration showed significant overall time effects (*p* = 0.012 and *p* = 0.024, respectively), whereas the remaining parameters did not reach statistical significance. These findings indicate that recovery following TKA is multidimensional, with significant changes in patient-reported outcomes, muscle strength, and functional mobility. iTUG analysis identified phase-specific changes, while several turning and transitional parameters did not show significant longitudinal changes, supporting the use of targeted rehabilitation strategies focusing on dynamic stability and movement control.

## 1. Introduction

KOA is a highly prevalent degenerative joint disease and a major cause of pain, disability, and reduced quality of life among older adults, with prevalence steadily increasing worldwide [1,2]. For individuals with end-stage KOA, TKA remains the most effective intervention, providing substantial pain relief, improved joint alignment, and restoration of mobility [3,4]. Despite these benefits, evidence indicates that gait abnormalities—such as reduced cadence, prolonged stride duration, asymmetry, and impaired rotational control—frequently persist for months or even years after surgery, thereby limiting full recovery and increasing fall risk [5,6,7].

Traditional gait analysis methods using motion capture and force platforms are considered the gold standard but remain costly and impractical for routine clinical follow-up. Wearable IMUs offer a valid and accessible alternative, enabling precise monitoring of spatiotemporal and kinematic gait parameters in real-world settings [8]. Among functional tests, the TUG test is widely recognized as a reliable screening tool for mobility and fall risk [9]. When instrumented with IMUs (iTUG), the test allows phase-specific quantification of accelerations, angular velocities, and turning performance, thereby capturing subtle recovery dynamics that are not detectable by stopwatch timing alone [10,11,12].

Several studies and systematic reviews have investigated the use of wearable sensors for gait and functional assessment following TKA [13,14,15,16,17,18]. However, substantial methodological heterogeneity exists regarding sensor technology, assessment protocols, follow-up periods, and reported outcomes [17,18]. Representative longitudinal studies are summarized in Table 1. Previous studies have used wearable sensors to characterize postoperative mobility and gait recovery across different follow-up periods, including instrumented TUG assessment and real-world gait monitoring [19,20,21]. However, these studies have primarily focused on sensor-derived mobility or gait outcomes, whereas the simultaneous integration of patient-reported outcomes, muscle strength, and phase-specific iTUG parameters has received less attention.

The novelty of the present study lies in its integrative, longitudinal design, combining the KOOS5 composite score, isometric muscle strength testing, and detailed iTUG-derived spatiotemporal, acceleration, and rotational parameters. Our aim was to characterize recovery trajectories across multiple domains following TKA and to determine how changes in gait and neuromuscular performance align with subjective perceptions of function and quality of life. This may improve clinical decision-making and individualized rehabilitation strategies.

## 2. Materials and Methods

### 2.1. Participants

**Figure 1:**
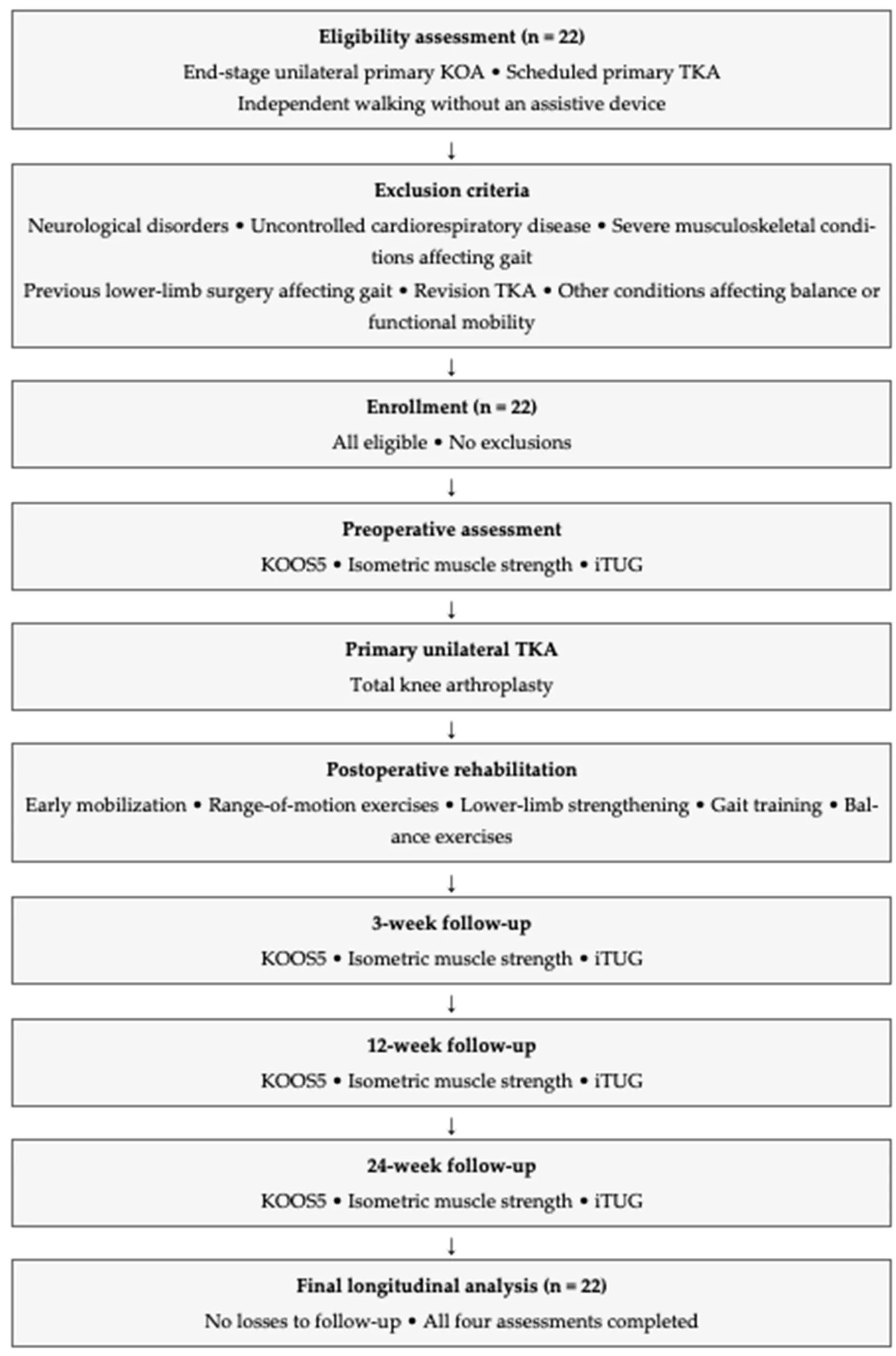
Flow chart of the study methodology, participant enrollment, and longitudinal assessment schedule.

In this prospective study, twenty-two patients diagnosed with end-stage unilateral primary KOA and scheduled for primary TKA were assessed for eligibility. All 22 patients met the eligibility criteria and were enrolled in the study, with no exclusions. Participants were 61–85 years old and were assessed preoperatively and at 3, 12, and 24 weeks postoperatively. All 22 participants completed all four assessments, with no losses to follow-up, and were included in the final repeated-measures analyses. The study methodology and participant flow are summarized in Figure 1.

The baseline demographic and anthropometric characteristics included age, height, body weight, shoe size, and body mass index (BMI). The eligibility criteria included a clinical and radiological diagnosis of end-stage unilateral primary KOA, scheduled primary TKA, and the ability to walk independently without an assistive device during testing. The exclusion criteria comprised neurological disorders, uncontrolled cardiorespiratory disease, severe musculoskeletal conditions affecting gait, previous lower limb surgery substantially influencing gait, revision TKA, and any other condition affecting balance or functional mobility.

All participants provided written informed consent prior to enrollment. The study was conducted in accordance with the Declaration of Helsinki and approved by the Institutional Review Board of the School of Physical Education and Sport Science, National and Kapodistrian University of Athens (approval no. 1306/22-09-2021).

### 2.2. Surgical Procedure and Postoperative Rehabilitation

All participants underwent primary unilateral TKA via a standard medial parapatellar approach executed by the same surgical team. Postoperatively, the patients followed a standard rehabilitation protocol, including early mobilization, range-of-motion exercises, lower limb strengthening, gait training, and balance exercises. However, rehabilitation frequency and progression were not standardized across participants or controlled by the investigators. Therefore, rehabilitation exposure may have differed between participants and contributed to variability in the observed outcomes.

### 2.3. Outcome Measures

#### 2.3.1. Patient-Reported Outcome Assessment

Knee-related symptoms and function were assessed using the KOOS, a 42-item questionnaire comprising five subscales: Symptoms, Pain, Activities of Daily Living, Sport and Recreation Function, and Knee-related Quality of Life [22]. Each subscale was scored according to the official KOOS guidelines and transformed to a 0–100 scale, with higher scores indicating better knee-related health. For longitudinal analyses, a KOOS5 composite score was calculated as the unweighted mean of the five normalized, equally weighted subscale scores. The KOOS5 was used to provide a single patient-reported outcome and to reduce multiple statistical comparisons [23]. Assessments were performed preoperatively and at 3, 12, and 24 weeks.

#### 2.3.2. Isometric Muscle Strength Assessment

The maximal voluntary isometric strength of the hip abductors, hip adductors, hip flexors, knee flexors, and knee extensors of the operated limb was measured using a handheld dynamometer (K-Force Muscle Controller, Kinvent, Montpellier, France) [24,25]. All measurements were performed by the same examiner, using standardized testing procedures. The participants completed one familiarization trial before testing, followed by two maximal efforts for each muscle group. The best of the two valid trials was retained to represent maximal voluntary performance, consistent with the objective of assessing maximal isometric strength. Strength was recorded in newtons (N) and assessed preoperatively and at 3, 12, and 24 weeks.

#### 2.3.3. Instrumentation

Gait analysis was performed using the G-Walk system with a G-SENSOR 2 wireless inertial measurement unit (BTS Bioengineering, Milan, Italy), incorporating a triaxial accelerometer, gyroscope, and magnetometer. Data were acquired at 100 Hz, with accelerometer and gyroscope ranges of ±2 g and ±2000°/s, respectively, in accordance with the manufacturer’s settings for the TUG protocol. The IMU was secured with a semi-elastic belt over the L4 vertebral level, immediately above the iliac crest. Before each recording, with the sensor secured in position, participants remained still for several seconds while the G-Studio software performed its software-guided calibration/initialization procedure. Data acquisition commenced only after the software indicated that this procedure was complete and prompted the examiner to begin the test. Data were collected indoors on a level, non-metallic surface and processed using G-Studio software (version 1.3.0; BTS Bioengineering, Milan, Italy). Phase segmentation and outcome extraction were performed automatically by the proprietary G-Studio software, and the automatically identified phase boundaries were not manually modified. The manufacturer’s available documentation does not specify the filter type or cutoff frequencies, the specific phase-detection algorithm, or the event-detection thresholds used for TUG signal processing.

Previous studies have evaluated selected measurement properties of the G-Walk system. In healthy adults, test–retest reliability ICCs ranged from 0.85 to 0.99 for spatiotemporal gait parameters, while agreement with GAITRite was high for gait speed, cadence, stride length, and stride duration (ICCs = 0.88–0.97) [26]. However, agreement was lower for some gait phase durations [26,27]. For the instrumented TUG, excellent agreement with an optoelectronic system was reported for total test duration in patients with Parkinson’s disease (single-measure ICC = 0.996) [28]. These findings do not establish the validity of individual iTUG phase durations, acceleration measures, or rotational velocities in patients following TKA.

### 2.4. Instrumented Timed Up and Go Protocol

The IMU was secured with a semi-elastic belt over the L4 vertebral level, immediately above the iliac crest. The participants performed the iTUG test by standing up from a chair, walking 3 m at a self-selected speed, turning, returning to the chair, and sitting down, following a standardized verbal command. One familiarization trial was completed before the data collection. Two valid trials were recorded for each assessment, and the best valid trial was retained to represent the participant’s optimal functional performance under standardized testing conditions. Trials were repeated if recording errors or participant-related interruptions were observed.

### 2.5. Signal Processing and Outcome Variables

The recorded IMU signals were transferred wirelessly and processed using G-Studio software (version 1.3.0, BTS Bioengineering, Milan, Italy). The iTUG trial was methodically segmented into six distinct phases: sit-to-stand, forward gait, mid-turn, return gait, end-turn, and stand-to-sit phases. The following outcome measures were extracted.

- Overall performance: total analysis duration (s).
- Transitions, specifically sit-to-stand and stand-to-sit movements, were characterized by phase duration (seconds) and peak accelerations in the anteroposterior, mediolateral, and vertical directions (m/s^2^).
- Turning phases (mid-turn and end-turn): phase duration (s), maximum rotational velocity (°/s), and average rotational velocity (°/s).

### 2.6. Statistical Analysis

Data were analyzed using IBM SPSS Statistics (version 29.0; IBM Corp., Armonk, NY, USA). Continuous variables are expressed as mean (standard deviation). Normality was assessed using the Shapiro–Wilk test.

The study was designed to explore longitudinal recovery across multiple functional domains, and no single primary outcome was prespecified. Outcomes were evaluated within three domains: patient-reported knee function (KOOS5), isometric muscle strength, and iTUG-derived functional measures, including temporal, acceleration, and rotational parameters.

Longitudinal changes in clinical, strength, and gait outcomes across the four assessment time points were evaluated using a one-way repeated-measures analysis of variance (ANOVA). Mauchly’s test was used to assess sphericity, and Greenhouse-Geisser corrections were applied when necessary. For the iTUG-derived outcomes, significant main effects were followed by Bonferroni-adjusted pairwise comparisons across assessment time points. No additional multiplicity adjustment was applied across the different outcome variables. Statistical significance was set at *p* < 0.05.

### 2.7. Generative Artificial Intelligence Statement

During the manuscript preparation, generative artificial intelligence tools were used to assist with language editing and the organization of selected sections. These tools were not used to generate, analyze, or modify the study data. All AI-assisted content was critically reviewed and revised by the authors, who took full responsibility for the accuracy and integrity of the manuscript.

## 3. Results

### 3.1. Participant Characteristics

The sample had a mean age of 73.3 (6.52) years (range, 61–85). The average body weight was 83.45 kg with a standard deviation of 16.42 kg, and the range was 52–110 kg. The average height was 167.64 cm with a standard deviation of 9.59 cm, and the range was 150–184 cm. The mean shoe size was 40.27 (2.54) EU (range: 36–45 EU). The mean BMI was 29.7 (4.5) kg/m^2^ (range: 19.5–40.5 kg/m^2^). The sample included six men (27.3%) and 16 women (72.7%) (Table 2).

### 3.2. Patient-Reported Outcome

Clinical status and functional recovery were assessed using the KOOS5 composite score. The results are summarized in Table 3.

**Figure 2:**
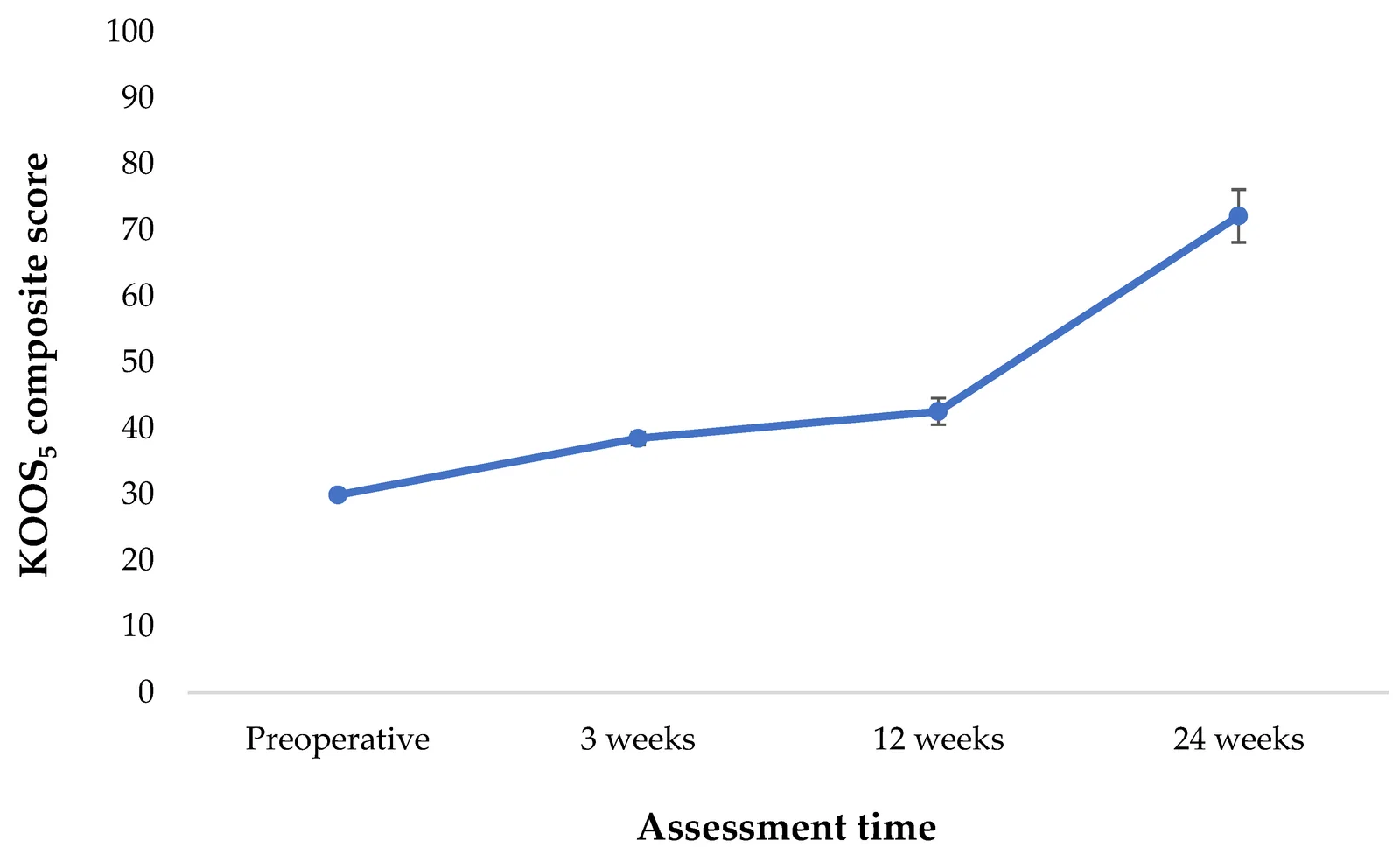
Longitudinal changes in the KOOS5 composite score following TKA.

The KOOS5 composite score showed a significant overall change over time (*p* < 0.001). Mean scores increased from 29.94 (12.67) preoperatively to 38.50 (11.27) at 3 weeks, 42.57 (19.56) at 12 weeks, and 72.17 (12.70) at 24 weeks (Figure 2).

### 3.3. Isometric Muscle Strength

**Figure 3:**
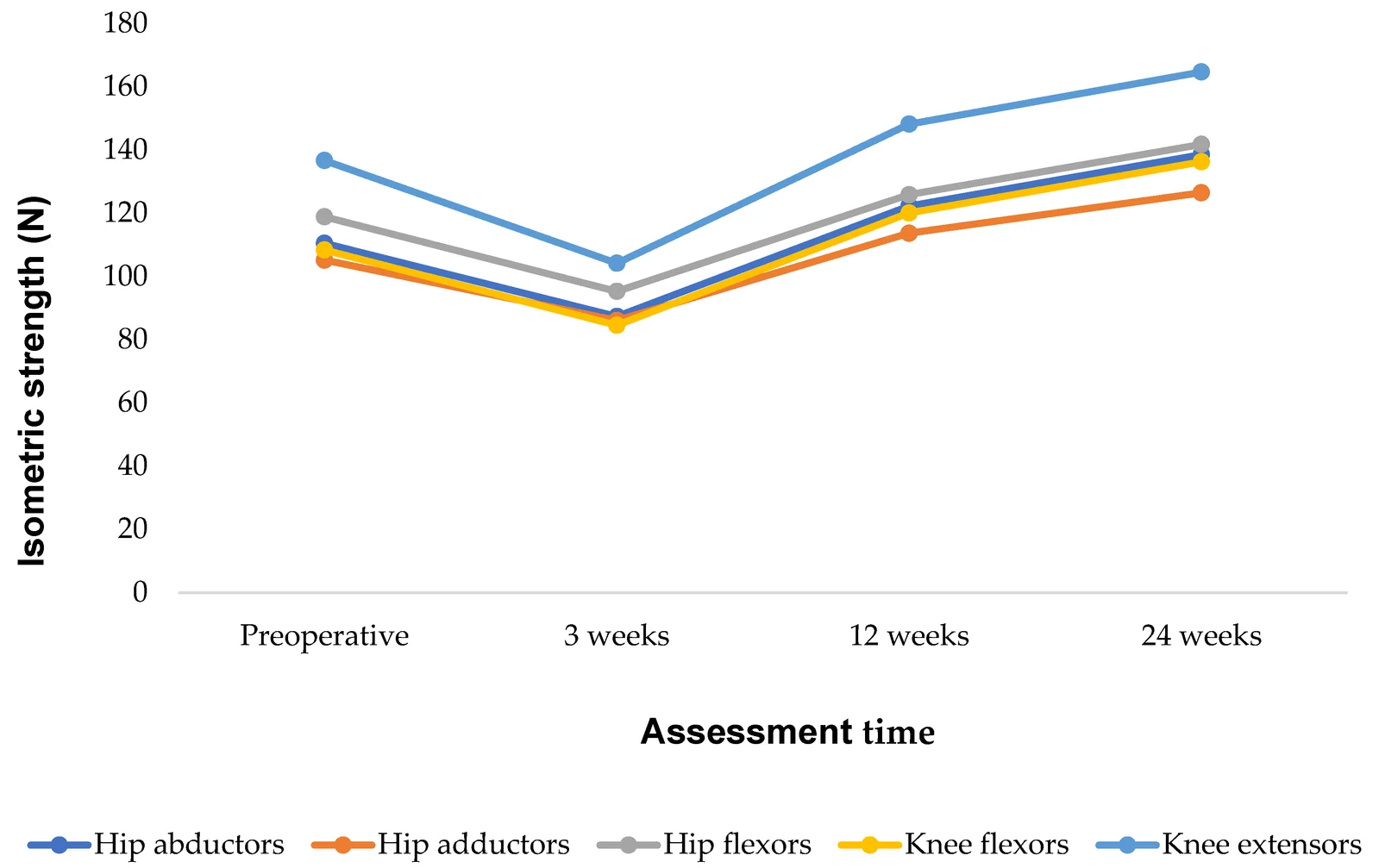
Longitudinal changes in isometric muscle strength following TKA.

Isometric muscle strength showed significant overall changes over time in all five muscle groups (Table 4). Hip abductor strength showed a significant overall time effect (*p* = 0.003), with mean values decreasing at 3 weeks and subsequently increasing through 24 weeks. Similar patterns in mean values were observed for the hip adductors (*p* = 0.008), hip flexors (*p* = 0.004), knee flexors (*p* = 0.002), and knee extensors (*p* = 0.001) (Figure 3).

### 3.4. Gait Analysis

Gait parameters during the TUG test were assessed preoperatively and at postoperative follow-up. The results are shown in Table 5.

The gait performance during the instrumented Timed Up and Go (iTUG) test is summarized in Table 5. Total analysis duration showed a significant overall effect of time (*p* = 0.023), decreasing from 22.26 (6.38) s preoperatively to 15.36 (3.63) s at 12 weeks, followed by a slight increase to 16.21 (4.89) s at 24 weeks. Bonferroni-adjusted post hoc analysis identified a significant reduction between the preoperative assessment and 12 weeks after surgery.

Significant improvements were also observed in the forward (*p* = 0.012) and return (*p* = 0.001) gait phases. Bonferroni-adjusted pairwise comparisons demonstrated a significant reduction in forward gait duration at 24 weeks compared with baseline, whereas return gait duration was significantly reduced at both 12 and 24 weeks postoperatively.

No significant changes were observed in the sit-to-stand, stand-to-sit, or turning phase durations (all *p* > 0.05), although most temporal parameters demonstrated numerical reductions over the postoperative period.

Among the kinematic variables, end-turning maximum rotational speed increased significantly over time (*p* = 0.012), with Bonferroni-adjusted post hoc analysis confirming a significant increase between baseline and 24 weeks. Sit-to-stand vertical acceleration also showed a significant overall effect of time (*p* = 0.024), with post hoc analysis demonstrating a significant increase between baseline and 12 weeks (*p* = 0.036). No significant changes were observed in the remaining acceleration or rotational variables (all *p* > 0.05), despite numerical changes across several measures.

## 4. Discussion

This study investigated longitudinal recovery following TKA by integrating patient-reported outcomes, isometric muscle strength, and iTUG performance using wearable inertial sensors. The findings indicate that recovery within the first six months following surgery is characterized by multiple dimensions. Significant overall changes were observed in patient-reported knee function, muscle strength, and objective mobility. However, recovery was not uniform across all functional domains, as several transitional and rotational gait parameters remained unchanged, suggesting that the restoration of complex motor control may follow a different recovery trajectory from changes in subjective function and muscle strength.

Regarding patient-reported outcomes, the KOOS_5_ composite score showed a significant overall change over time, with mean values increasing across the postoperative assessments. These findings are consistent with those of previous studies demonstrating that TKA effectively reduces pain and enhances daily function, with meaningful recovery occurring within the first six months after surgery [29]. However, improvements in patient-reported outcomes should not be interpreted as evidence of complete functional recovery. Pain relief following TKA often precedes the full restoration of neuromuscular function, and patients may perceive considerable improvements despite persistent objective functional deficits [29,30,31]. This highlights the complementary role of objective biomechanical assessments in identifying residual functional deficits that may not be captured by patient-reported questionnaires alone.

With respect to muscle performance, isometric strength showed significant overall changes over time in all evaluated muscle groups, with mean values demonstrating an early postoperative decline followed by subsequent increases. The initial reduction aligns with postoperative pain, swelling, arthrogenic muscle inhibition, and temporary decreases in voluntary muscle activation, which are typically observed following TKA [32]. By 24 weeks, mean strength values were higher than preoperative values, with particularly marked increases observed in the hip abductor and adductor muscles. Recovery of proximal hip muscle strength is clinically relevant because these muscles contribute to frontal plane pelvic stability, mediolateral balance, and efficient weight transfer during gait. Improved hip strength may reduce compensatory movement strategies and enhance overall walking stability during postoperative recovery [33].

Knee extensor and flexor strength also showed significant overall changes over time, with mean values increasing after the initial postoperative decline. The observed increases in mean strength values relative to preoperative levels do not inherently signify normalization when compared with healthy individuals. Studies have shown that quadriceps weakness may persist for over six months after TKA, despite significant clinical improvements [32]. This finding implies that extended strengthening programs may be necessary to improve muscle strength. Collectively, these findings reinforce the importance of early rehabilitation strategies targeting both quadriceps activation and proximal hip musculature to optimize functional recovery and support a safe and efficient gait following TKA [32,34].

Regarding functional mobility, iTUG analysis revealed significant improvements in overall performance, primarily driven by shorter forward and return gait phases. These findings indicate progressive restoration of walking efficiency and are consistent with previous reports demonstrating improvements in spatiotemporal gait characteristics during the first six months after TKA [17]. In contrast, no significant changes were observed in the sit-to-stand, stand-to-sit, or turning phase durations, suggesting that the recovery of more demanding motor tasks may occur more slowly than that of straight-line walking.

This difference is biomechanically plausible because of the increased neuromuscular coordination required for turning and transitional movements, as opposed to level walking. Successful turning involves rapid deceleration and acceleration, coordinated trunk and lower limb rotation, anticipatory postural adjustments, and continuous regulation of the center of mass while maintaining dynamic balance [35]. Similarly, sit-to-stand and stand-to-sit transfers require substantial lower limb strength, postural control, and precise coordination. Consequently, despite significant improvements in walking speed and muscle strength, complex functional tasks may remain impaired [36]. In this context, the absence of significant changes in the duration of these phases may reflect a slower recovery of neuromuscular control and dynamic postural stability compared with straight-line walking. Furthermore, the variability observed in these more complex movement phases may have reduced the ability to detect statistically significant changes over the six-month follow-up period.

Among the kinematic variables, end-turning maximum rotational velocity and sit-to-stand vertical acceleration showed significant changes over time. These findings indicate changes in movement dynamics during postoperative recovery; however, in the absence of established clinical reference values or thresholds, higher rotational velocity or vertical acceleration should not necessarily be interpreted as improved movement quality or functional performance. Rather, these parameters may provide complementary information regarding changes in movement characteristics that are not captured by temporal measurements alone. Although the remaining acceleration and rotational variables did not reach statistical significance, numerical changes were observed across several measures; however, their clinical significance remains uncertain and requires further investigation.

From a clinical perspective, these findings emphasize that postoperative recovery should not be evaluated solely using the total TUG time or patient-reported outcomes. Improvements in overall mobility may coexist with a lack of significant longitudinal changes in several turning and transitional parameters. Although falls were not assessed in the present study, previous research in patients following TKA has associated persistent balance and mobility deficits with an increased risk of falls [37,38]. Previous evidence also supports the assessment of turning as a distinct dimension of mobility in individuals undergoing TKA [14]. Longitudinal data indicate that walking and turning may follow different recovery trajectories after TKA, with improvements in several walking parameters while turning velocity may show limited or no significant change over time [39]. Consequently, rehabilitation following TKA should extend beyond conventional gait retraining and incorporate exercises targeting multidirectional mobility, dynamic balance, proprioception, and coordinated rotational movements to optimize functional recovery.

A significant strength of this study is the use of wearable inertial measurement units to objectively quantify recovery. Unlike conventional stopwatch-based TUG assessment, instrumented analysis enables the separate evaluation of the gait, turning, and transfer phases, together with acceleration and rotational characteristics. This phase-specific approach provided a more comprehensive understanding of postoperative recovery and identified phase-specific changes in functional performance that would not have been detected using the total test duration alone. As wearable sensor technology becomes increasingly accessible in clinical practice, instrumented functional assessments may support individualized rehabilitation by enabling clinicians to monitor recovery more precisely and to identify patients who require targeted interventions.

Overall, the present findings demonstrate that recovery following TKA is multidimensional and progresses at different rates across the functional domains. While substantial improvements were observed in patient-reported outcomes, muscle strength, and walking performance, several parameters related to more complex motor tasks, particularly turning and transitional movements, did not show statistically significant longitudinal changes during the first six postoperative months. These results highlight the importance of combining patient-reported measures with objective sensor-based gait analysis to obtain a comprehensive evaluation of recovery and guide individualized rehabilitation strategies following TKA [8,17].

## 5. Limitations and Future Directions

This study had several limitations. First, the relatively small sample size may have limited the detection of subtle but clinically relevant differences and may have reduced the generalizability of the findings. Second, the absence of a healthy control group precluded direct comparison with normative gait patterns and limited the ability to determine whether postoperative improvements represented complete functional recovery or persistent compensatory movement strategies. Third, although wearable inertial measurement units (IMUs) provide objective assessments of spatiotemporal, acceleration, and rotational gait parameters, they do not quantify joint-level kinematics, kinetics, or muscle activation patterns. These additional measurements could provide further insights into the mechanisms underlying recovery. Moreover, the cited validation studies do not establish the measurement validity of phase-specific iTUG accelerations and rotational velocities in patients following TKA. These variables should therefore be interpreted cautiously. Furthermore, rehabilitation frequency and progression were not standardized across participants, and detailed information regarding rehabilitation duration and adherence was not systematically recorded for individual participants. Therefore, between-participant variability in rehabilitation exposure could not be quantified. These differences may have contributed to variability in muscle strength and functional mobility over time. Consequently, the observed changes reflect postoperative recovery following TKA under the rehabilitation conditions received; the contribution of rehabilitation cannot be distinguished from other recovery-related factors. Additionally, the use of the best of two valid trials for strength and iTUG outcomes may have favored optimal rather than typical performance. Future studies may consider averaging repeated valid trials to provide an estimate of typical performance and reduce potential trial-selection bias. Because multiple outcomes were examined without adjustment for multiplicity across outcome variables, the possibility of type I error cannot be excluded. Accordingly, these findings should be interpreted as exploratory and require confirmation in larger studies. The follow-up period was restricted to 24 weeks, which precluded the evaluation of long-term recovery beyond the first six postoperative months.

Future studies should include larger multicenter cohorts with longer follow-up periods to improve external validity and characterize recovery beyond six months after total knee arthroplasty. Incorporating healthy control participants would enable comparison with normative gait patterns and facilitate assessment of the degree to which postoperative gait normalizes over time. In addition, integrating wearable IMUs with motion capture, force platform analysis, and electromyography could provide a more comprehensive understanding of the biomechanical and neuromuscular mechanisms underlying recovery. Further validation of phase-specific iTUG acceleration and rotational velocity measures in patients following TKA is also needed. Finally, prospective studies evaluating standardized rehabilitation protocols, particularly those targeting muscle strength, dynamic stability, proprioception, and turning performance, may help optimize functional recovery after total knee arthroplasty.

## 6. Conclusions

In summary, significant overall changes over time were observed in functional mobility, gait performance, muscle strength, and patient-reported outcomes during the first six postoperative months. The integration of wearable sensor-based TUG analysis with clinical and subjective measures provides a comprehensive view of recovery, highlighting both substantial changes and differing recovery patterns across functional mobility parameters. These findings support the value of combining objective and subjective assessments in postoperative monitoring and emphasize the need for rehabilitation strategies targeting advanced neuromuscular control and stability. Future longitudinal studies should extend the follow-up period beyond six months, incorporate healthy control groups, and evaluate targeted rehabilitation interventions designed to optimize long-term neuromuscular recovery and functional mobility following TKA.

## Figures and Tables

**Table 1:** Comparison of representative wearable sensor-based studies of functional and gait recovery following total knee arthroplasty with the present study.

Study	Design/Sample	Wearable Assessment	Follow-Up	Main Outcomes/Focus
Kwasnicki et al., 2015 [19]	Longitudinal feasibility study; 14 TKA patients + 15 healthy controls	Ear-worn motion sensor (e-AR); TUG and functional mobility tasks	Preoperative; 1, 3, 6, 12, and 24 weeks	Sensor-derived functional mobility and postoperative recovery profiling
Bloomfield et al., 2019 [20]	Longitudinal; 68 primary unilateral TKA patients	Wearable sensor–instrumented TUG	Preoperative; 2, 6, and 12 weeks	Multiple sensor-derived TUG metrics and early functional recovery patterns
Ghaffari et al., 2025 [21]	Prospective longitudinal; 31 unilateral TKA patients	Thigh-mounted accelerometers; continuous real-world gait monitoring	2 weeks preoperative to 90 days postoperative	Temporal and frequency-domain gait features combined with patient-reported outcomes
Present study	Prospective longitudinal; 22 TKA patients	G-Walk IMU; phase-specific iTUG + isometric muscle strength + KOOS5	Preoperative; 3, 12, and 24 weeks	Integrated longitudinal assessment of patient-reported function, muscle strength, and phase-specific temporal, acceleration, and rotational iTUG parameters

**Table 2:** Baseline characteristics of the study participants [mean (standard deviation) and range].

Parameter	Patients with Knee OA	Range
Age (years)	73.3 (6.52)	61–85
Body weight (kg)	83.45 (16.42)	52–110
Height (cm)	167.64 (9.59)	150–184
Shoe size (EU)	40.27 (2.54)	36–45
BMI (kg/m^2^)	29.7 (4.5)	19.5–40.5
Sex	6 men (27.3%), 16 women (72.7%)	—

**Table 3:** Longitudinal changes in the KOOS5 composite score following TKA [mean (standard deviation)].

Questionnaire	Preoperative	3 Weeks	12 Weeks	24 Weeks	Overall *p*-Value
KOOS5 composite score	29.94 (12.67)	38.50 (11.27)	42.57 (19.56)	72.17 (12.70)	<0.001

**Table 4:** Recovery of isometric muscle strength pre- and post-TKA [mean (standard deviation)].

Muscle Group	Preoperative	3 Weeks	12 Weeks	24 Weeks	Overall *p*-Value
Hip abductors	110.5 (21.2)	87.4 (18.3)	122.3 (20.9)	138.6 (22.1)	0.003
Hip adductors	105.2 (20.6)	86.1 (19.7)	113.7 (21.4)	126.5 (23.0)	0.008
Hip flexors	118.9 (23.1)	95.3 (20.5)	125.9 (24.8)	141.8 (25.2)	0.004
Knee flexors	108.4 (19.8)	84.6 (18.2)	120.1 (20.7)	136.3 (21.5)	0.002
Knee extensors	136.7 (26.5)	104.2 (24.1)	148.2 (25.7)	164.7 (26.9)	0.001

**Table 5:** Gait parameter analysis during the TUG test in patients following TKA. Values are presented as mean (standard deviation).

Parameter	Preoperative	3 Weeks	12 Weeks	24 Weeks	Overall *p*-Value
Analysis duration (s)	22.26 (6.38)	17.48 (2.64)	15.36 (3.63)	16.21 (4.89)	0.023
Sit-to-stand phase duration (s)	2.35 (0.68)	2.05 (0.51)	1.88 (0.24)	1.78 (0.55)	0.140
Stand-to-sit phase duration (s)	2.27 (0.55)	2.13 (0.61)	2.37 (0.39)	2.60 (0.86)	0.597
Forward gait phase duration (s)	5.90 (2.54)	4.00 (0.90)	3.38 (1.36)	3.17 (0.82)	0.012
Return gait phase duration (s)	6.08 (2.40)	3.44 (0.20)	3.11 (0.98)	2.87 (0.74)	0.001
Mid-turning phase duration (s)	2.82 (0.87)	3.52 (1.41)	2.36 (0.50)	3.00 (2.21)	0.502
End-turning phase duration (s)	3.12 (0.86)	2.53 (0.44)	2.39 (0.63)	2.87 (1.59)	0.355
Sit-to-stand anteroposterior acceleration (m/s^2^)	2.55 (1.16)	2.50 (1.07)	2.84 (0.95)	3.27 (1.72)	0.645
Stand-to-sit anteroposterior acceleration (m/s^2^)	2.94 (1.52)	4.53 (1.68)	3.59 (1.40)	4.12 (2.74)	0.327
Sit-to-stand lateral acceleration (m/s^2^)	1.39 (0.59)	1.48 (0.78)	2.29 (1.06)	1.75 (0.58)	0.077
Stand-to-sit lateral acceleration (m/s^2^)	3.11 (1.90)	3.53 (2.26)	3.57 (0.84)	3.92 (2.03)	0.805
Sit-to-stand vertical acceleration (m/s^2^)	2.78 (1.01)	3.15 (0.75)	4.49 (1.90)	4.12 (1.29)	0.024
Stand-to-sit vertical acceleration (m/s^2^)	4.63 (2.62)	7.43 (1.35)	5.96 (3.21)	6.73 (4.77)	0.308
Mid-turning maximum rotation speed (°/s)	118.83 (36.12)	101.48 (17.60)	143.97 (19.27)	153.07 (48.37)	0.062
End-turning maximum rotation speed (°/s)	105.69 (34.37)	141.80 (22.88)	137.41 (28.04)	161.80 (48.79)	0.012
Mid-turning average rotation speed (°/s)	59.99 (19.88)	51.73 (15.44)	71.71 (11.40)	70.35 (34.07)	0.365
End-turning average rotation speed (°/s)	53.20 (16.24)	67.23 (10.69)	68.46 (17.59)	70.85 (30.29)	0.154

## Data Availability

The data presented in this study are included in the article. Additional information is available from the corresponding author upon reasonable request.

## Abbreviations

The following abbreviations are used in this manuscript:

TKA	Total knee arthroplasty
KOA	Knee osteoarthritis
TUG	Timed Up and Go
iTUG	Instrumented Timed Up and Go
IMUs	Inertial measurement units
KOOS	Knee Injury and Osteoarthritis Outcome Score

## References

[1] Hunter D.J., Schofield D., Callander E. (2014). The Individual and Socioeconomic Impact of Osteoarthritis. Nat. Rev. Rheumatol..

[2] Chen J., Chen X., Wang T., Li M., Dai H., Shang S., Cheng L., Tang Z., Liu S., Huang C. (2025). Global Burden of Knee Osteoarthritis from 1990 to 2021: Trends, Inequalities, and Projections to 2035. PLoS ONE.

[3] Goh S.L., Persson M.S.M., Stocks J., Hou Y., Welton N.J., Lin J., Hall M.C., Doherty M., Zhang W. (2019). Relative Efficacy of Different Exercises for Pain, Function, Performance and Quality of Life in Knee and Hip Osteoarthritis: Systematic Review and Network Meta-Analysis. Sports Med..

[4] Choi K.Y.H., Chau W.W., Li L.M.K., Ng S.Y.L., Lo B.P.Y., Ong M.T.Y., Yung P.S.H. (2025). Improving Pain, Function and Quality of Life in End-Stage Knee Osteoarthritis: A Patient-Preference Cohort Study on Whole-Body Vibration and Exercise as Bridging Therapies for Total Knee Replacement. Arthroplasty.

[5] Schache M.B., McClelland J.A., Webster K.E. (2014). Lower Limb Strength Following Total Knee Arthroplasty: A Systematic Review. Knee.

[6] Zeni J.A., Flowers P., Bade M., Cheuy V., Stevens-Lapsley J., Snyder-Mackler L. (2019). Stiff Knee Gait May Increase Risk of Second Total Knee Arthroplasty. J. Orthop. Res..

[7] Zolkoske A.C., Fehr S.D. (2024). Gait Disturbances. Nelson Pediatric Symptom-Based Diagnosis: Common Diseases and Their Mimics.

[8] Picerno P., Iosa M., D’Souza C., Benedetti M.G., Paolucci S., Morone G. (2021). Wearable Inertial Sensors for Human Movement Analysis: A Five-Year Update. Expert Rev. Med. Devices.

[9] Podsiadlo D., Richardson S. (1991). The Timed Up and Go: A Test of Basic Functional Mobility for Frail Elderly Persons. J. Am. Geriatr. Soc..

[10] Bragonzoni L., Rovini E., Barone G., Cavallo F., Zaffagnini S., Benedetti M.G. (2019). How Proprioception Changes before and after Total Knee Arthroplasty: A Systematic Review. Gait Posture.

[11] Buisseret F., Catinus L., Grenard R., Jojczyk L., Fievez D., Barvaux V., Dierick F. (2020). Timed up and Go and Six-Minute Walking Tests with Wearable Inertial Sensor: One Step Further for the Prediction of the Risk of Fall in Elderly Nursing Home People. Sensors.

[12] Risitano S., Cacciola G., Capella M., Bosco F., Giustra F., Fusini F., Indelli P.F., Massé A., Sabatini L. (2023). Comparison between Gaits after a Medial Pivot and Posterior Stabilized Primary Total Knee Arthroplasty: A Systematic Review of the Literature. Arthroplasty.

[13] Tao W., Liu T., Zheng R., Feng H. (2012). Gait Analysis Using Wearable Sensors. Sensors.

[14] Boekesteijn R.J., van Gerven J., Geurts A.C.H., Smulders K. (2022). Objective Gait Assessment in Individuals with Knee Osteoarthritis Using Inertial Sensors: A Systematic Review and Meta-Analysis. Gait Posture.

[15] Gianzina E., Yiannakopoulos C.K., Armenis E., Chronopoulos E. (2025). Wearable Sensor Assessment of Gait Characteristics in Individuals Awaiting Total Knee Arthroplasty: A Cross-Sectional, Observational Study. J. Funct. Morphol. Kinesiol..

[16] Gianzina E., Yiannakopoulos C.K., Kalinterakis G., Delis S., Chronopoulos E. (2025). Evaluation of the Timed Up and Go Test in Patients with Knee Osteoarthritis Using Inertial Sensors. Int. J. Transl. Med..

[17] Gianzina E., Kalinterakis G., Delis S., Vlastos I., Platon Sachinis N., Yiannakopoulos C.K. (2023). Evaluation of Gait Recovery after Total Knee Arthroplasty Using Wearable Inertial Sensors: A Systematic Review. Knee.

[18] Feng Y., Liu Y., Fang Y., Chang J., Deng F., Liu J., Xiong Y. (2023). Advances in the Application of Wearable Sensors for Gait Analysis after Total Knee Arthroplasty: A Systematic Review. Arthroplasty.

[19] Kwasnicki R.M., Ali R., Jordan S.J., Atallah L., Leong J.J.H., Jones G.G., Cobb J., Yang G.Z., Darzi A. (2015). A Wearable Mobility Assessment Device for Total Knee Replacement: A Longitudinal Feasibility Study. Int. J. Surg..

[20] Bloomfield R.A., Williams H.A., Broberg J.S., Lanting B.A., McIsaac K.A., Teeter M.G. (2019). Machine Learning Groups Patients by Early Functional Improvement Likelihood Based on Wearable Sensor Instrumented Preoperative Timed-Up-and-Go Tests. J. Arthroplast..

[21] Ghaffari A., Clasen P.D., Kappel A., Rasmussen J., Gurchiek R.D., Kold S., Rahbek O. (2025). Monitoring Gait Recovery After Total Knee Arthroplasty Using Wearable Sensors: Responsiveness of Gait Accelerations. J. Orthop. Res..

[22] Roos E.M., Lohmander L.S. (2003). The Knee Injury and Osteoarthritis Outcome Score (KOOS): From Joint Injury to Osteoarthritis. Health Qual. Life Outcomes.

[23] Roos E.M. (2024). 30 Years with the Knee Injury and Osteoarthritis Outcome Score (KOOS). Osteoarthr. Cartil..

[24] Pippas C., Emmanouilidis A., Karanasios S., Koumantakis G., Gioftsos G., Pippas C., Emmanouilidis A., Karanasios S., Koumantakis G., Gioftsos G. (2024). Reliability of the K-Force Muscle Controller Dynamometer on Eccentric and Isometric Hip Adduction Strength. Open J. Ther. Rehabil..

[25] Taveira N., Fernandes A.I., Barbosa De Almeida M., Oliveira C., Ornelas G., Soares T., Souto J., Póvoa A.R., Maia L., Ferreira A. (2023). Intra-Rater and Inter-Rater Reliability of the Kinvent Hand-Held Dynamometer in Young Adults. Med. Sci. Forum.

[26] De Ridder R., Lebleu J., Willems T., De Blaiser C., Detrembleur C., Roosen P. (2019). Concurrent Validity of a Commercial Wireless Trunk Triaxial Accelerometer System for Gait Analysis. J. Sport Rehabil..

[27] Viteckova S., Horakova H., Polakova K., Krupicka R., Ruzicka E., Brozova H. (2020). Agreement between the GAITRite R System and the Wearable Sensor BTS G-Walk R for Measurement of Gait Parameters in Healthy Adults and Parkinson’s Disease Patients. PeerJ.

[28] Kleiner A.F.R., Pacifici I., Vagnini A., Camerota F., Celletti C., Stocchi F., De Pandis M.F., Galli M. (2018). Timed Up and Go Evaluation with Wearable Devices: Validation in Parkinson’s Disease. J. Bodyw. Mov. Ther..

[29] Bade M.J., Stevens-Lapsley J.E. (2012). Restoration of Physical Function in Patients Following Total Knee Arthroplasty: An Update on Rehabilitation Practices. Curr. Opin. Rheumatol..

[30] Wylde V., Beswick A., Bruce J., Blom A., Howells N., Gooberman-Hill R. (2018). Chronic Pain after Total Knee Arthroplasty. EFORT Open Rev..

[31] Stevens-Lapsley J.E., Schenkman M.L., Dayton M.R. (2011). Comparison of Self-Reported Knee Injury and Osteoarthritis Outcome Score to Performance Measures in Patients After Total Knee Arthroplasty. PM R.

[32] Paravlic A.H., Meulenberg C.J., Drole K. (2022). The Time Course of Quadriceps Strength Recovery After Total Knee Arthroplasty Is Influenced by Body Mass Index, Sex, and Age of Patients: Systematic Review and Meta-Analysis. Front. Med..

[33] Stocker R., Wittauer M., Kvarda P., Nüesch C., Appenzeller-Herzog C., Halbeisen F., Mündermann A., Ismailidis P. (2025). Hip Abductor Muscle Strength in Patients After Total Knee Arthroplasty: A Systematic Review and Meta-Analysis. J. Arthroplast..

[34] Singla R., Niederer D., Franz A., Happ K., Zilkens C., Wahl P., Behringer M. (2023). The Course of Knee Extensor Strength after Total Knee Arthroplasty: A Systematic Review with Meta-Analysis and -Regression. Arch. Orthop. Trauma Surg..

[35] Xu D., Carlton L.G., Rosengren K.S. (2004). Anticipatory Postural Adjustments for Altering Direction during Walking. J. Mot. Behav..

[36] Sadeh S., Gobert D., Shen K.H., Foroughi F., Hsiao H.Y. (2023). Biomechanical and Neuromuscular Control Characteristics of Sit-to-Stand Transfer in Young and Older Adults: A Systematic Review with Implications for Balance Regulation Mechanisms. Clin. Biomech..

[37] Moutzouri M., Gleeson N., Billis E., Tsepis E., Panoutsopoulou I., Gliatis J. (2017). The Effect of Total Knee Arthroplasty on Patients’ Balance and Incidence of Falls: A Systematic Review. Knee Surg. Sports Traumatol. Arthrosc..

[38] Chan A.C.M., Jehu D.A., Pang M.Y.C. (2018). Falls After Total Knee Arthroplasty: Frequency, Circumstances, and Associated Factors-A Prospective Cohort Study. Phys. Ther..

[39] Bruning F.J., Boekesteijn R.J., Defoort K.C., Susan S., van den Ende C.H.M., Smulders K. (2026). Recovery of Daily-Life Walking after Total Knee Arthroplasty: A Two-Year Longitudinal Study and Comparison with Healthy Controls. Osteoarthr. Cartil. Open.

